# miR-200b-3p relieved inflammation in patients with heart failure by regulating ZEB1 expression

**DOI:** 10.1186/s13019-024-02628-8

**Published:** 2024-05-03

**Authors:** Bo Wei, Zhiyong Li, Li Wang, Haitao Zhang, Wen Gou

**Affiliations:** Department of Cardiology, Yongchuan Hospital Affiliated of Chongqing Medical University, No. 439, Xuanhua Road, Yongchuan District, Chongqing, 402177 China

**Keywords:** Heart failure, MiR-200b-3p, ZEB1, Inflammatory response, ROC curve

## Abstract

**Background:**

MicroRNA-200b-3p (miR-200b-3p) plays a pivotal role in inflammatory responses and is implicated in various inflammatory disorders. In this study, we aim to explore the role of miR-200b-3p in the inflammatory response in heart failure (HF).

**Methods:**

Patients diagnosed with heart failure and age-matched healthy controls were studied. Peripheral blood samples from participants were collected for RNA-seq analysis to explore the expression profile of miR-200b-3p. The predictive value of miR-200b-3p and ZEB1 in the prognosis of heart failure was evaluated by analyzing the receiver operating characteristic (ROC) curve. Bioinformatics analysis and double luciferase reporter gene analysis were used to confirm the interaction between miR-200b-3p and ZEB1. Real-time quantitative polymerase chain reaction (QRT-PCR) was used to detect the expression levels of miR-200b-3p and ZEB1 in cardiopulmonary bypass. Additionally, the effects of miR-200b-3p on myocardial cell line (H9c2) injury were evaluated by enzyme-linked immunosorbent assay (ELISA).

**Results:**

In the extracardiac circulation of HF patients, miR-200b-3p expression was significantly reduced, while ZEB1 levels were notably elevated. Analysis of the ROC curve revealed that miR-200b-3p and ZEB1 have predictive value in the prognosis of HF patients. The double luciferase reporter experiment demonstrated that miR-200b-3p binds to ZEB1 and inhibits its expression. Overexpression of miR-200b-3p demonstrated a remarkable ability to alleviate inflammation and inhibit the damage to myocardial cells in vivo.

**Conclusion:**

MiR-200b-3p can target and inhibit ZEB1, reducing the inflammatory reaction of myocardial cells. The miR-200b-3p/ZEB1 network may be helpful in preventing and treating HF.

## Introduction

Heart failure (HF) is a prevalent global epidemic and a primary cause of sudden mortality. As the population ages, the incidence and prevalence of this condition continue to escalate [1, 2]. HF is a complicated condition that may be driven on by many factors, the most common of which include hypertension, age, diabetes, coronary heart disease, and obesity [3]. Over the last several decades, various treatment methods, such as pacing and defibrillation treatment, heart transplantation or mechanical assisted circulation support, have been widely used in HF [3]. However, insufficient research progress has been made on the pathophysiology and molecular mechanisms of HF, leading to a significant decrease in the quality of life for HF patients [4]. Consequently, gaining a comprehensive understanding of the intricate mechanisms underlying HF will help identify new targets and even expand the window for treating HF.

Numerous studies have illustrated that microRNAs (miRNAs) participated in various cellular processes. Within the miR-200 family, miR-200b-3p holds significant importance [5, 6]. Prior investigations have illustrated that miR-200b-3p participated in regulating the prognosis of HF [7, 8]. Nevertheless, the precise function of miR-200b-3p in the onset and progression of HF remains limited, especially its signal transduction pathway and its interaction with some mRNA in the prognosis of HF.

ZEB1, as a transcription regulatory factor, induces endometrial transformation and exerts a pivotal influence on both normal physiological and pathological processes [9]. It was originally recognized as a DNA-binding protein that encompasses a homology domain along with two clusters of zinc fingers, which facilitate its interaction with two high-affinity binding sites located in the E-cadherin promoter region, thereby regulating the biological function of cells [10]. Research has found that ZEB1 can regulate IL-6 and IL-8 protein production, promoting inflammatory response. Inflammation assumes a close relationship with acute and chronic HF, and multiple inflammatory mediators are crucial to the progression of HF and cardiovascular disease [11]. Despite this recognition, the regulatory impact of ZEB1 on cellular immune inflammation in HF remains insufficiently explored. Consequently, our study aims to clarify the regulating impact of miR-200b-3p on ZEB1, and further analyzed the biological role of this miRNA in the prognosis of HF.

## Materials and methods

### Main reagent

Myocardial cell line H9c2 (National Biomedical Experimental Cell Resource Bank, USA), DMEM medium (Gibco, USA), Fetal bovine serum (FBS, Hyclone, USA), X-tremeGENE9 (Roche, Switzerland), double luciferase reporter gene detection system kit (Sigma, Germany), overexpression of miR-200b-3p vector (Genechem, Shanghai), ELISA kit (Mlbio, Shanghai), Polyclonal antibodies (Abcam, USA), isoproterenol (Sigma, Germany).

### Collection of sample tissues

The subjects were 100 patients diagnosed with heart failure at Yongchuan Hospital of Chongqing Medical University. The inclusion criteria were as follows: (1) adults over 18 years old, (2) diagnosed with heart failure (clinically using the Framingham standard or echocardiography), and (3) received follow-up care in the heart clinic for at least three months. The exclusion criteria included severe respiratory diseases, chronic inflammation, organic heart disease, and hyperthyroidism. The normal control group consisted of 50 age-matched healthy subjects without cardiovascular disease or metabolic disorders. This study received approval from the Medical Ethics Committee of Yongchuan Hospital of Chongqing Medical University. The peripheral blood of the patients was collected and separated into serum and blood cells. The blood cells were stored at -80 °C after adding the appropriate RNAlater solution.

### RNA-seq analysis

RNA extraction from blood cells was performed using TRIzol reagent. Following quality inspection, a cDNA library was constructed. After passing the quality control, the Illumina HiSeq4000 platform was utilized for high-throughput sequencing. The sequencing process and result analysis were conducted by Beijing Nuohe Zhiyuan Company. Specifically, |log2(fold change, FC)| > 1 and *P* < 0.05 were used as cutoffs to screen differentially expressed genes. An online website was employed to create heat maps. The KOBAS software was utilized to conduct KEGG pathway enrichment analysis on the genes associated with each distinct set of differentially expressed transcripts, and the screening threshold for significant differences was set to *P* < 0.05.

### Cell culture and experimental grouping

H9c2 cardiomyocytes were cultured in DMEM medium containing 10% FBS and Penicillin-Streptomycin (80 U/ml) at 37 °C and 5% O2. For the experimental setup, four distinct groups were established: the control group, ISO group, ISO-miR-NC group, and ISO-miR-200b-3p group. The control group utilized culture medium as the control and added 50 µM ISO. Following the instructions of the X-tremeGENE9 kit, the expression vector was transfected into H9c2 myocardial cells. After 24 to 48 h of cell culture, well-growing cells were selected for subsequent experiments.

### QRT-PCR experiment

QRT-PCR was conducted following the protocols outlined in the SYBR fluorescence quantitative assay kit instructions. The QRT-PCR reaction was set for 40 cycles with an initial denaturation at 94 °C for 5 min, followed by denaturation at 94 °C for 20 s, annealing at 60 °C for 1 min, and signal collection at 60 °C. To perform relative quantitative analysis, internal references such as GAPDH and U6 were utilized. The 2^−ΔΔCT^ method was employed for data analysis in this study.

### Bioinformatics prediction and double luciferase experiment

The binding sites of miR-200b-3p and ZEB1 were predicted using the online gene prediction website StarBase database. These cells were divided into four groups, namely ZEB1 (WT) + miR-NC group, ZEB1 (WT) + miR-200b-3p group, ZEB1 (MUT) + miR-NC group, and ZEB1 (MUT) + miR-200b-3p group. The dual luciferase reporter vectors were transfected into cells, and cell lysate lysis and 10 µL LAR II reagent were added. As an internal reference, sea kidney luciferase was utilized, and the firefly luciferase activity was measured using an enzyme labeling instrument.

### Western blot experiment

Total protein was extracted from cell samples. The protein concentration was detected using the BCA kit. Load 40 µg protein samples per lane (used for detecting NLRP3 and ZEB1) were separated using 10% SDS-PAGE. Under the action of an electric field, proteins were transferred to the PVDF membrane. Seal the membrane with skim milk, dilute the primary antibody to a certain volume, and incubate it overnight at 4 °C. After washing, incubate with the corresponding secondary antibody for 2 h, and expose the target band in a chemiluminescent solution. Quantitative analysis of protein bands was performed using ImageJ software. Calculate the relative quantification of proteins using GAPDH as a control.

### ELISA experiment

Dilute the standard substance and detect antibodies according to the method described in the ELISA kit. Add cell supernatant and incubate for 1 h. Subsequently, add the enzyme-labeled reagent and incubate for 50 min. Next, add the substrate chromogenic agent and incubate for 15 min. Finally, terminate the reaction, gently shake, zero with a blank, and measure the OD values at 450 nm of each well in sequence using a microplate reader. Calculate the levels of various inflammatory factors based on the standard curve and sample OD values.

### Statistical analysis

SPSS 22.0 software was utilized to analyze all experimental data. Continuous data were represented using the format mean ± standard deviation (x ± s). To compare between two groups, we employed the t-test. For comparing multiple groups, we utilized one-way ANOVA. Finally, for pairwise comparisons, we employed the LSD-T test. We consider *P* < 0.05 to represent a statistically significant difference.

## Results

### RNA-seq analysis of HF patients and normal controls

**Figs. 1:**
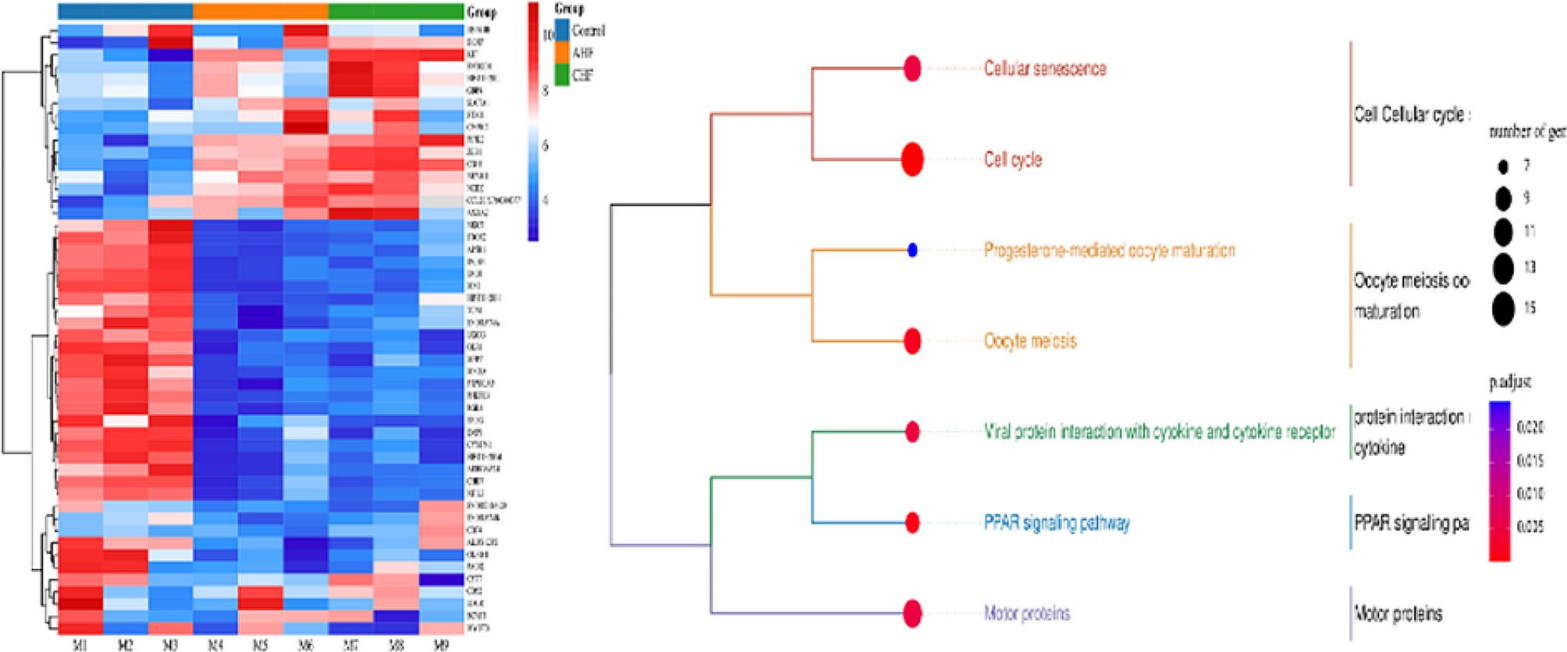
RNA-seq analysis of HF patients and normal controls. mRNA differential expression heatmap (left) and KEGG evolutionary tree map (right)

According to the different courses of HF patients, they were divided into acute HF (AHF) and chronic HF (CHF). The overall hierarchical clustering diagram of differential mRNA is shown in Fig. 1. Fifty-one differentially expressed mRNAs were analyzed and screened, of which 16 were upregulated and 35 were downregulated in the blood of HF patients. The upregulated genes include KIT, SLC40A1, ENDOD1, NFAM1, CD45, and ZEB1. KEGG enrichment analysis was carried out for mRNA candidate target genes, among which the most significant 7 pathways were: cell cycle, oocyte meiosis and maturation, protein interaction and cytokines, PPAP signal pathway, and motor protein (Fig. 1).

### Bioinformatics prediction and validation of miR-200b-3p bound to ZEB1

**Figs. 2:**
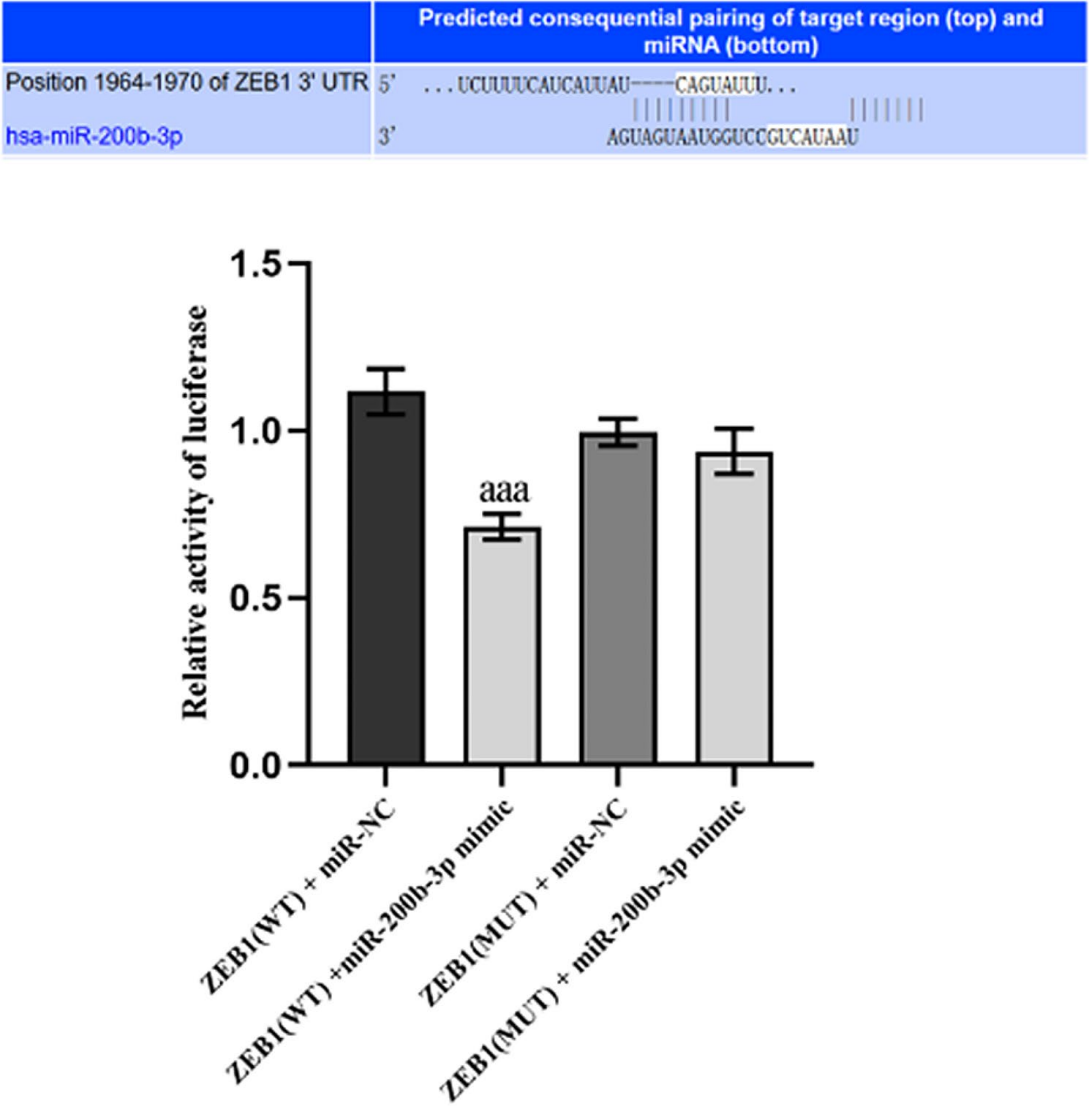
miR-200b-3p directly targeted ZEB1. Binding sites between miR-200b-3p and ZEB1; The relative luciferase activity. aaa: *P* < 0.001, compared to the ZEB1 (WT) + miR-NC group

We further predicted the potential binding sites between miR-200b-3p and ZEB1 using the online bioinformatics StarBase database. The prediction results suggest the presence of a potential miR-200b-3p binding site in the 3′UTR of ZEB1. In comparison to the ZEB1 (WT) + miR-NC group, the relative luciferase activity was significantly reduced in the ZEB1 (WT) + miR-200b-3p group, indicating a significant inhibition of luciferase activity in the ZEB1 (WT) + miR-200b-3p group. Notably, no significant difference was observed between the ZEB1 (MUT) + miR-200b-3p and ZEB1 (MUT) + miR-NC groups (Fig. 2).

### Expression and prognostic values of miR-200b-3p and ZEB1 in HF

**Figs. 3:**
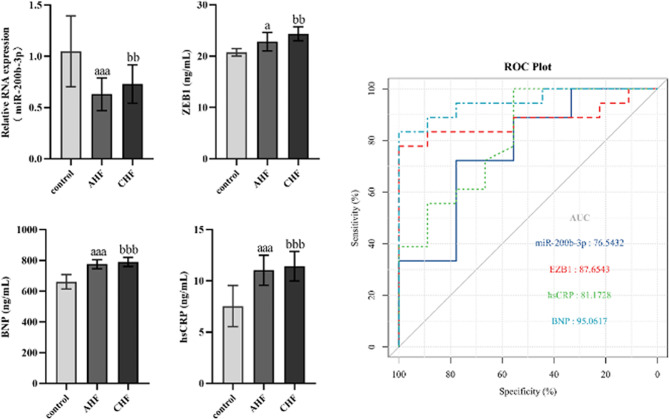
Expression and prognostic values of miR-200b-3p, ZEB1, BNP, and hsCRP in HF patients. AHF showed: a: *P* < 0.05, aaa: *P* < 0.001; CHF showed: bb: *P* < 0.01, bbb: *P* < 0.001, compared to the control group

To examine the levels of miR-200b-3p, ZEB1, BNP, and hsCRP among the control group, AHF group, and CHF group, qPCR and ELISA were used to detect the peripheral blood of HF patients. In the AHF and CHF groups, miR-200b-3p expression was significantly reduced compared to the control group. Furthermore, HF patients exhibited significantly elevated levels of ZEB1, BNP, and hsCRP. To further assess the predictive efficacy of these four genes on HF patients, we constructed ROC curves and subsequently computed the AUC values. ROC analyses demonstrated that the AUC values of miR-200b-3p, ZEB1, BNP, and hsCRP for HF patients were 0.654, 0.8765, 0.8117, and 0.9506, respectively (Fig. 3).

### Influence of miR-200b-3p on ZEB1 expression in H9c2 cardiomyocytes

**Figs. 4:**
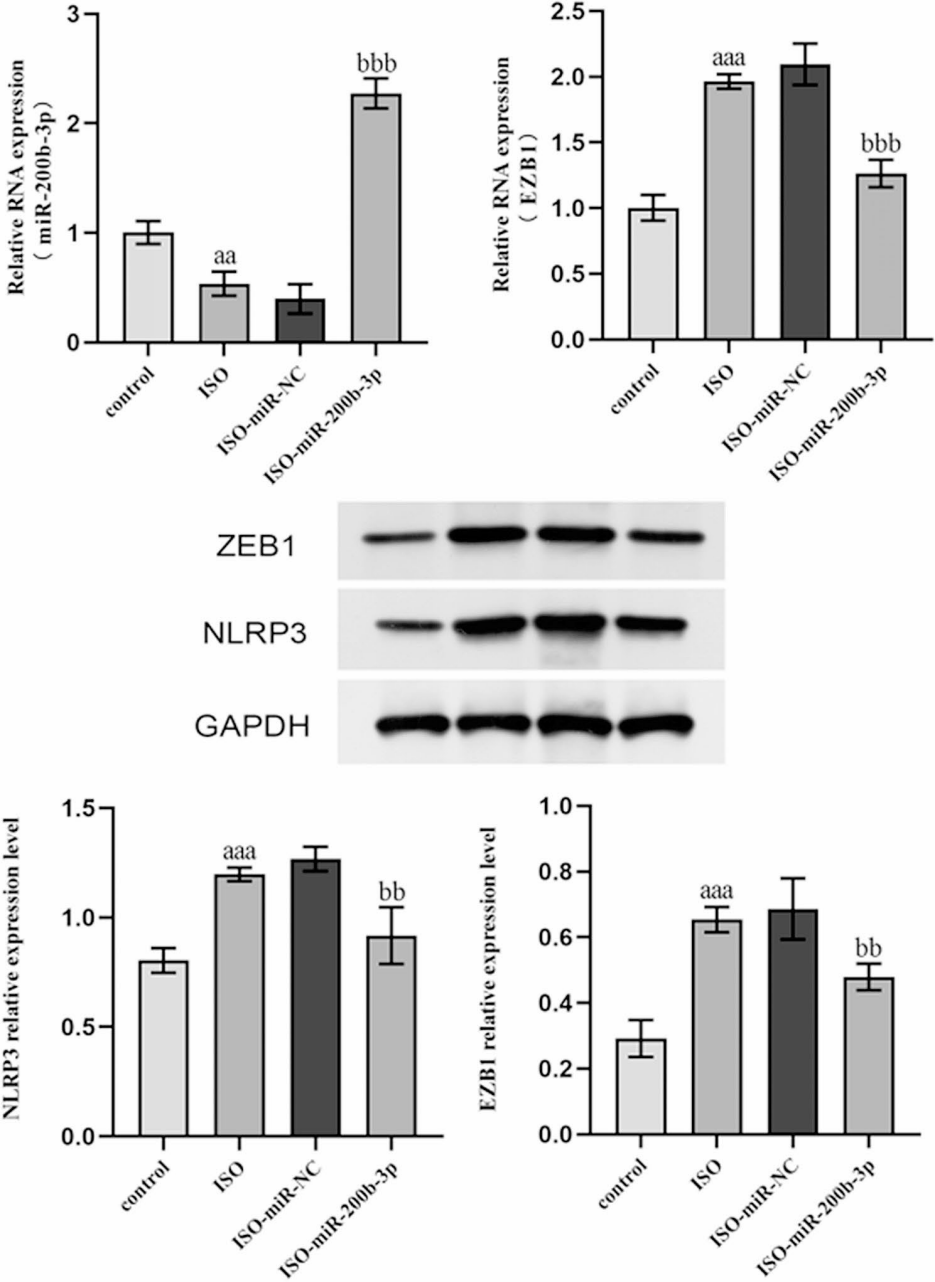
Influence of miR-200b-3p on ZEB1 expression. aa: *P* < 0.01, aaa: *P* < 0.00, compared to the ISO and control groups; bb: *P* < 0.01, bbb: *P* < 0.001, compared to the ISO-miR-200b-3p group

We further transfected the miR-200b-3p overexpression vector into ISO-induced H9c2 cells. Upon ISO induction, miR-200b-3p expression was remarkably decreased, while ZEB1 expression and NLRP3 expression were remarkably increased compared to the control group. Following transfection with the overexpression vector, compared to the ISO-miR-NC group, we observed a remarkable elevation in miR-200b-3p expression and a notable reduction in ZEB1 expression and NLRP3 expressions in the ISO-miR-200b-3p group (Fig. 4).

### MiR-200b-3p regulates inflammatory factors in H9c2 cardiomyocytes

**Figs. 5:**
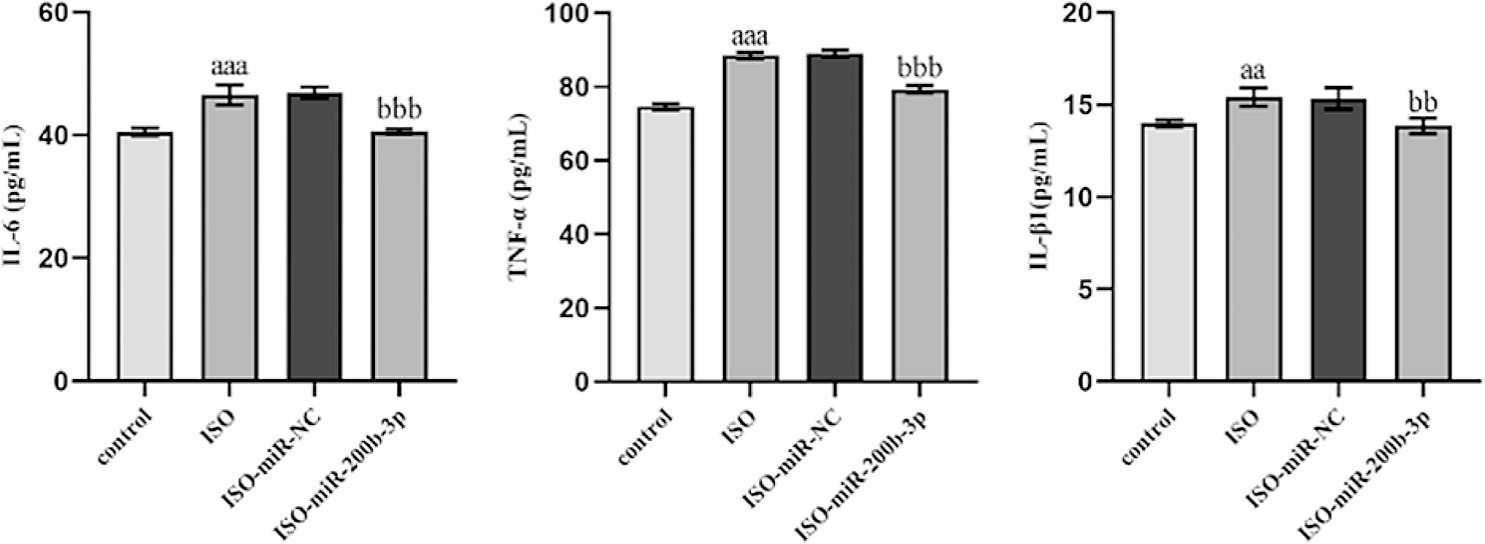
Effect of miR-200b-3p on inflammatory factors in ISO-induced H9c2 cardiomyocytes. aa: *P* < 0.01, aaa: *P* < 0.00, compared to the control group; bb: *P* < 0.01, bbb: *P* < 0.001, compared to the ISO miR-200b-3p group

To elucidate the role of miR-200b-3p transfection on inflammatory factors in H9c2 cardiomyocytes induced by ISO, the levels of inflammatory factors in cells were detected using ELISA. Compared with the control group, cells induced by ISO exhibited a substantial increase in IL-6, IL-β1, and TNF-α levels. Notably, the overexpression of miR-200b-3p remarkably attenuated the levels of these three proinflammatory factors in comparison to the ISO-miR-NC group (Fig. 5).

## Discussion

Heart failure (HF) cases are on the rise, and the absence of efficient therapies significantly impacts the associated mortality rate [12]. Recently, a large number of studies have highlighted the highly connected relationship between miRNAs and the progression of HF, suggesting their potential as therapeutic targets [13, 14]. Research has found that heart-related miRNAs protect the heart from the effects of pathological hypertrophy and HF by directly targeting downstream genes [15]. While the association between miR-200b-3p and various human diseases is well-known, its specific impact on HF remains ambiguous and is receiving increasing attention. In the present study, we aim to thoroughly clarify the function and mechanisms of miR-200b-3p on HF patients and ISO-induced H9c2 cardiomyocytes. Notably, our findings demonstrate a significant reduction in miR-200b-3p expression in HF patients, suggesting a potential protective effect on HF.

Several investigations have validated the applicability of miR-200b as a biomarker for assessing visceral fibrosis in HF [7]. Additionally, several genes that directly target miR-200b-3p were discovered, such as HLF, SMAD2, TIMP4, and Notch1 [16–19]. At the same time, miR-200b-3p targeting ZEB1 inhibits EMT and participates in cancer invasion and migration. C-reactive protein (CRP) is believed to exacerbate myocardial injury, and the concentrations of hsCRP and BNP are independently associated with HF and cardiovascular risk [20]. Hence, we detected levels of miR-200b-3p, ZEB1, hsCRP, and BNP in HF patients. Our findings demonstrated a notable downregulation in miR-200b-3p expression, while the expression of ZEB1, hsCRP, and BNP exhibited a notable upregulation. Moreover, employing clinical predictive models, we demonstrated that miR-200b-3p, ZEB1, hsCRP, and BNP hold significant predictive value in determining the prognosis of HF patients.

ZEB1 can activate endoplasmic reticulum stress signal transduction and block induced apoptosis, cardiomegaly, and HF [21]. In our investigation, we revealed upregulation of NFAM1, CD45, and ZEB1 in the peripheral blood of HF patients using RNA-seq. KEGG enrichment analysis showed significant enrichment in signaling pathways, including the cytokines and PPAP pathway. ZEB1 is an important downstream target of miR-200b-3p, which has also been validated in our experiments. According to reports, ZEB1 can promote the release of inflammatory mediators and promote the progression of myocardial injury [22]. To elucidate the effects of miR-200b-3p targeting ZEB1 on the inflammatory response mechanism in cardiomyocytes, we performed miR-200b-3p overexpression experiments in H9c2 cardiomyocytes. Our experimental outcomes confirmed that miR-200b-3p overexpression resulted in a reduction in ZEB1 expression and decreased levels of inflammatory factors.

## Conclusion

In summary, our study confirms the downregulation of miR-200b-3p expression and the upregulation of ZEB1 expression in patients with HF. Furthermore, we have identified that miR-200b-3p binds to ZEB1, leading to a negative regulation of its expression. Additionally, miR-200b-3p overexpression exhibits a notable ability to reduce the release of inflammatory factors, thereby attenuating the inflammatory response of myocardial cells. Hence, the miR-200b-3p/ZEB1 pathway emerges as a promising therapeutic approach for HF.

## Data Availability

All data generated or analysed during this study are included in this. Further enquiries can be directed to the corresponding author.

## Abbreviations

HF: Heart failure
miRNAs: microRNAs
FC: Fold change
FBS: Fetal bovine serum
AHF: Acute HF
CHF: Chronic HF
CRP: C-reactive protein
